# Supplementary material for: Heparin Inhibits SARS-CoV-2 Replication in Human Nasal Epithelial Cells
Source: Viruses. 2022 Nov 24;14(12):2620. doi: 10.3390/v14122620 (PMC9785945; doi:10.3390/v14122620)
Supplement: Supplementary file 1 [file viruses-14-02620-s001.zip › viruses-2011340-supplementary.pdf]

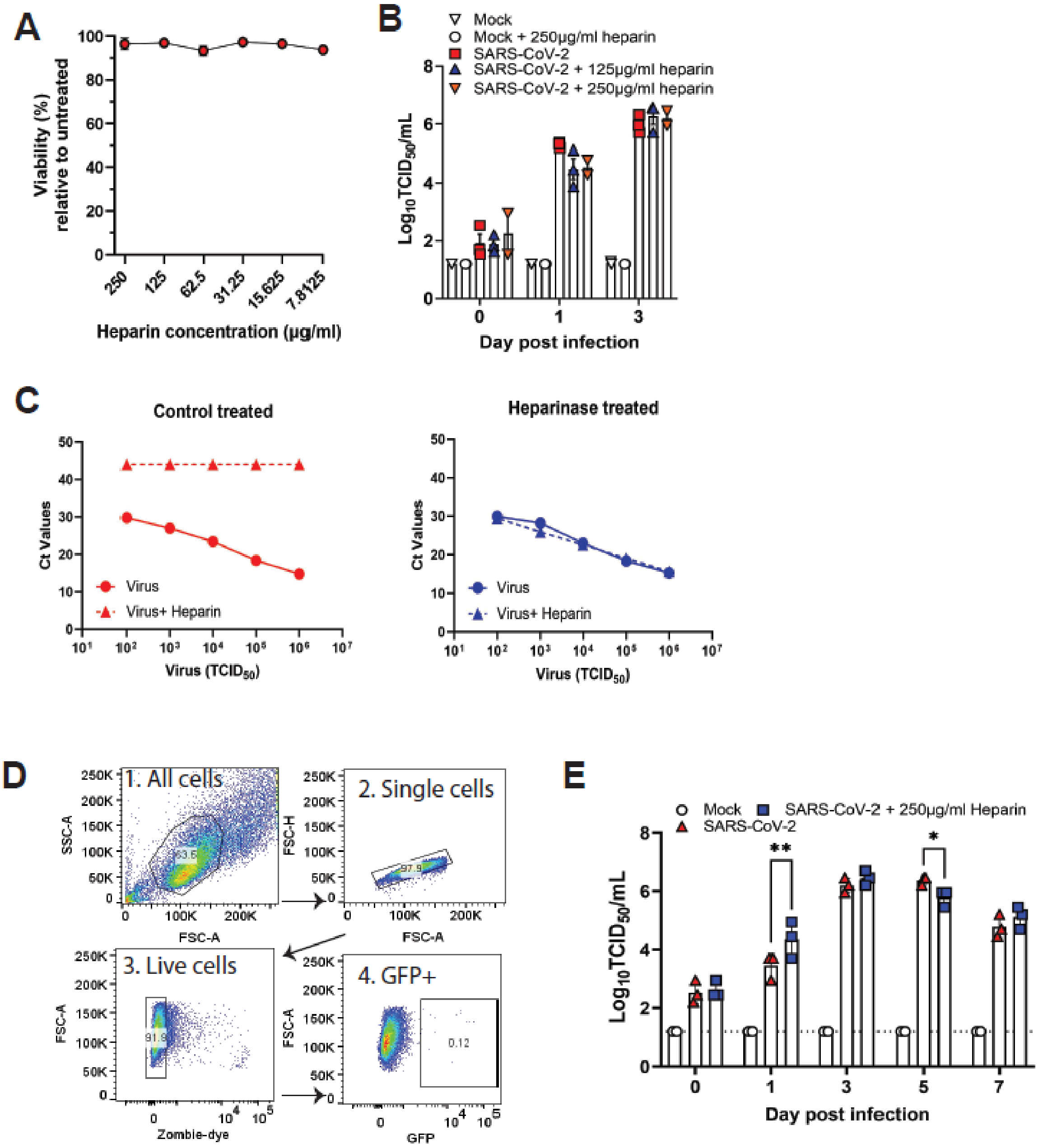

Figure S1. (**A**) Viability of Vero cells treated with indicated heparin concentrations for 3 days. Graph is a representative of 2 experiments each with 3 replicates per concentration. (**B**) Infectious virus titers in supernatants fromVero cells infected with SARS-CoV-2 (MOI 0.02) and cultured with or without heparin for 3 days. Graph shows the mean titer +/− SEM of 3 replicates from 3 separate experiments, except for 250 µg/mL that was tested in 2 experiments. (**C**) Cycle threshold (CT) values of SARS-CoV-2 E-gene from indicated concentrations of SARS-CoV-2 virus with or without 250µg/mL of heparin. Isolated RNA was treated with 0.5 units of heparinase or an equivalent volume of buffer and RNaseOUT before RT-PCR analysis. (**D**) Flow cytometry gating strategy. (**E**) Infectious virus titers in apical supernatants from human nasal epithelial cells infected with virus incubated with or without heparin for 1 h prior to infection. Heparin was not present through-out the experiment. Graph shows the mean +/− SEM of 3 replicates from 1 donor. Data were analyzed using a Two-Way ANOVA with Dunnett's multiple comparisons test. *, $p < 0.05$ and **, $p > 0.01$.
